# Multiple-Point Temperature Gradient Algorithm for Ring Laser Gyroscope Bias Compensation

**DOI:** 10.3390/s151229777

**Published:** 2015-11-30

**Authors:** Geng Li, Pengfei Zhang, Guo Wei, Yuanping Xie, Xudong Yu, Xingwu Long

**Affiliations:** College of Opto-electrics Science and Engineering, National University of Defense Technology, Changsha 410073, China; lg_163@163.com (G.L.); zhangpengfei0309@163.com (P.Z.); nudtweiguo@163.com (G.W.); xyp99999@139.com (Y.X.); wind0909@163.com (X.Y.)

**Keywords:** error compensation, particle swarm optimization, ring laser gyroscope, support vector machine, gradient methods, temperature sensors, temperature measurement

## Abstract

To further improve ring laser gyroscope (RLG) bias stability, a multiple-point temperature gradient algorithm is proposed for RLG bias compensation in this paper. Based on the multiple-point temperature measurement system, a complete thermo-image of the RLG block is developed. Combined with the multiple-point temperature gradients between different points of the RLG block, the particle swarm optimization algorithm is used to tune the support vector machine (SVM) parameters, and an optimized design for selecting the thermometer locations is also discussed. The experimental results validate the superiority of the introduced method and enhance the precision and generalizability in the RLG bias compensation model.

## 1. Introduction

The ring laser gyro (RLG) has become the current mainstream rotation rate sensor in the high-precision strap-down inertial navigation system (SINS), which is widely used in military and commercial applications. It has many advantages, such as digital output, a stable scale factor, temperature and vibration reliability, and is also lightweight [1,2]. However, in actual commercial or military application environments, the variation of temperature within the device contributes to an uneven RLG bias error and degrades the SINS performance. In order to solve this problem, many researchers have conducted in-depth and careful investigations to improve the environmental adaptability utilizing different compensation models.

The traditional and most convenient model is the least-square fitting (LSF) algorithm [3,4,5,6]. By utilizing the least-square error criterion, the LSF model compensates for the RLG bias by computing the coefficients of a hypothetical polynomial. The advantage of the LSF model is its short run time, which is especially useful in real-time systems without high-precision requirements. For SINS used in high-accuracy, long working time, real-time output applications such as the high-altitude long-endurance unmanned aerial vehicle (HALE UAV), submarines, and land vehicles, an RLG bias temperature compensation model with wide suitability and higher accuracy is necessary.

Nonlinear modeling methods such as artificial neural networks (ANNs) [7,8,9,10,11,12] and support vector machines (SVMs) [13,14,15,16] have been utilized in the temperature modeling of RLG bias compensation in recent years. For a complex nonlinear system, an ANN has an excellent ability of approximating a system through network training. In particular, the back-propagation neural network (BPNN) has greatly improved the RLG bias precision by properly fitting the temperature-related variables [7]. Moreover, the radial-basis-function neural network (RBFNN) has a better compensation result and shorter computing time than BPNN [8,9,10], because the average number of iterations of RBFNN is less than that of BPNN. However, under variable temperature environments, traditional RBFNN has many limitations, such as poor generalizability and lengthy computation times [11]. To overcome these shortcomings, a modified RBFNN was introduced based on the Kohonen network and the orthogonal least squares (OLS) algorithm [12]. Compared to multiple linear regression (MLR) and traditional RBFNN, the RLG bias compensation result with the modified RBFNN achieved higher accuracy and required less computational time. However, in the model training stage, the empirical risk minimization (ERM) principle was employed, which could lead to over-fitting or under-fitting, compromising local optimization.

To resolve these problems, RLG bias compensation models utilizing SVM have been developed as substitutes. Least-square SVMs (LS-SVMs) used in the system-level temperature compensation of RLG have effectively reduced the temperature variation influence on the RLG bias and improved the accuracy [15]. By choosing the proper parameters (penalty factor and kernel width), LS-SVM converts the quadratic optimization problem into a problem requiring the solution of linear equations, which reduces the computational complexity.

Moreover, to simultaneously enhance the precision and generalizability, a method based on the particle swarm optimization-support vector machine (PSO-SVM) has been proposed, which has multiple temperature variable inputs [16]. The PSO [17,18] is a advanced machine learning algorithm which is broadly used in many fields, such as multidimentional knapsack problem (MKP) [19,20], economic and statistical design [21], and complex network reliability problem [22]. Filtered by the adaptive forward-linear prediction (FLP) algorithm, the noise in the RLG bias data with temperature are filtered and preprocessed. Then, the SVM model of RLG bias compensation utilizing the PSO algorithm is employed to guarantee precision and generalizability. The temperature sampling data are obtained using three thermometers, which are attached to the anode, cathode, and shell of the RLG. The thermometers selection is based on the analysis in [3]. In [3], the thermometer positions are selected according to the theoretical analysis and experiment; the characteristics of the whole temperature field are simplified using four points: the cathode, anode, angular rate sensor, and cabinet. However, by utilizing only four points, the optimization conclusion does not accurately reflect the complex temperature field of the working RLG.

To understand the complete temperature field characteristics of the working RLG, a thermal finite-element method (FEM) simulation of the working RLG is developed in this study. Then, the whole working RLG temperature field is divided into several small sections, which are equipped with thermometers, to describe the RLG temperature characteristics in detail. The temperature, temperature variation, and temperature gradient parameters are discussed in order to assess the effectiveness of a specific section or of multiple sections in the RLG by utilizing correlation coefficients with the RLG bias. After finding the most effective input temperature parameters for the RLG bias compensation under the different temperature variation rates, in order to improve the model development, the PSO-SVM model is established based on the optimal model parameters. Finally, two experiments are conducted: one is set based on regular temperature variation conditions for model training and verification, while the other is set based on random temperature variation conditions for model testing. The experimental results show that the critical temperature point for the RLG bias compensation is the cathode area temperature. The temperature gradients between this temperature point and the other temperature points show the most correlation with the RLG bias. Combined with the PSO-SVM model, the RLG bias compensation using the optimal temperature gradients achieves high accuracy under random temperature changing conditions.

This paper is organized as follows. In Section 2, the temperature characteristics of the working RLG are simulated with FEM, and the multiple-point temperature compensation model is constructed and analyzed for RLG bias. In Section 3, the principles of the SVM model and PSO algorithm for optimal modeling of the RLG temperature bias are described in detail. In Section 4, the temperature experiments are executed in order to refine and validate the proposed method, and the results are analyzed in detail. The conclusions are presented in Section 5.

## 2. Multiple-Point Temperature Compensation Analysis of RLG Bias

The temperature characteristics of a working RLG are very complicated, because the RLG integrates optical, electronic, and machinery components with different types of heat sources in an unpredictable thermal environment. To comprehend the temperature characteristics of the working RLG, in general, we first perform a FEM simulation. The main heat sources of the RLG include three parts: (1) the resonant cavity between the RLG cathode and the two anodes, which is filled with mixed He-Ne gases; (2) the pre-amplifier circuit, which is equipped in the cabinet and converts the photodiode signals to proper voltage signals; and (3) two ballast resistances, which are attached near the two anodes. For other components, such as the piezoelectric (PZT) actuator used for the path length control (PLC) system and the dither mechanism, the mechanical energy of which are converted to thermal energy, the heat output is very small. Therefore, these components can be neglected in the FEM model for the purpose of concision. The thermal parameters of each major material in the RLG assembly are listed in Table 1.

**Table 1:** Thermal parameters of materials in ring laser gyro (RLG) assembly.

Material	Density (kg/m^3^)	Specific Heat (J/kg·K)	Thermal Conductivity (W/m·K)
Zerodur crystallite glass	2530	800	1.46
Ballast resistances	3700	386	398
Pre-amplifier Circuit	2140	386	398

When the thermal characteristics of the RLG are analyzed, the main sources of heat are equivalent to the models with different heat generation rates (HGENs), which are shown in Table 2 [23].

**Table 2:** The heat source in RLG assembly model.

Heat Source	Equivalent Work (W)	Volume × 10^6^ (m^3^)	Heat Generation Rates × 10^4^ (W/m^3^)
Gain area	0.588	1.126	52.22
Preamplifier circuit	0.784	35	2.24
Ballast resistance	0.027	0.295	24.41

**Figure 1:**
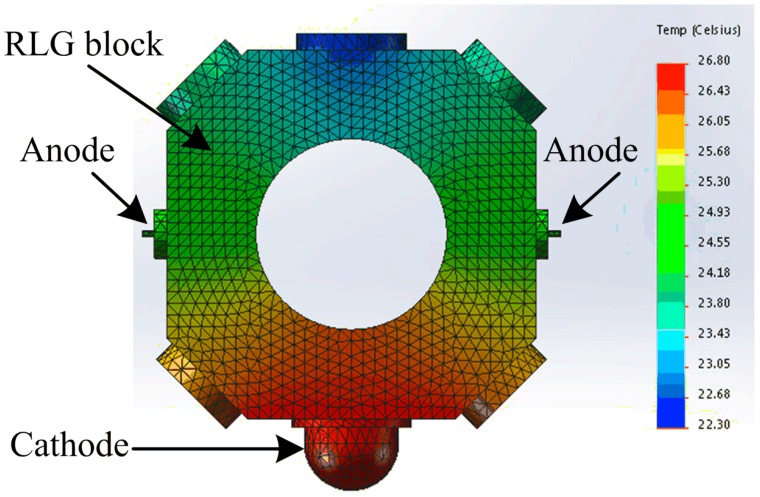
Thermal image of working RLG.

The simulation is performed in the ANSYS Workbench software by using the steady-state thermal analysis tool. The initial ambient temperature is set to 20 °C; the 20-node thermal element SOLID90 is used in the RLG block model, which is divided into 198,908 elements (including 304,562 nodes), and the RLG block steady-state temperature field is shown in Figure 1.

**Figure 2:**
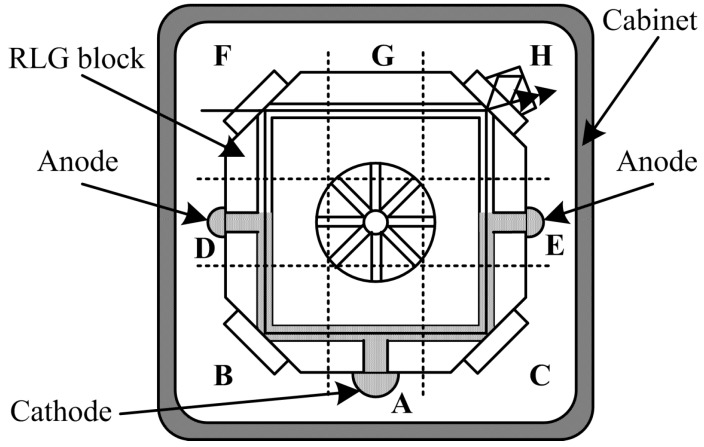
Thermometer arrangement in RLG.

According to Figure 1, during the RLG operation, the gain area between the cathode and the two anodes reaches a higher temperature than the nongain area, which is defined as the area in the opposite face of the gain area. The average difference in temperature between the gain and nongain areas is 3.5 °C. The two arms in the gain area should ideally have the same temperature; however, there are still some residual differences, because of variations in the anode material, ballast resistance, and so on. Therefore, to describe the thermo-field of the full-scale working RLG, as many thermometers as possible are placed in the RLG in accordance with the following principles: (1) the temperature should reflect the gain medium working condition, which means that the temperature surrounding the optic resonant cavity should be measured; (2) the extreme temperature conditions of the RLG and cabinet shell should be considered, including the highest and lowest temperature; (3) the temperatures of the two arms of the gain area should be measured symmetrically, because the difference in temperatures between the arms will lead to an imbalance in the gas flow inside the optic resonator; this may eventually cause an RLG bias error; and (4) the environmental temperature of the RLG should be measured, because it is the main cause of the gradient in temperature that influences the RLG bias. Based on these rules, thermometers A to H are arranged in the RLG as shown in Figure 2.

As shown in Figure 2, the space in the RLG cabinet is divided into nine areas: area A is near the RLG cathode; areas B and C are symmetrically located at the corners of the RLG surrounding the cathode; areas D and E are near the two RLG anodes; and areas F, G, and H are located opposite the cathode areas. Because the RLG has symmetric construction and because mechanical dither, which is located at the center of the RLG, contributes little to the heat generation, the center area is not considered in the simulation and temperature experiment. The areas A, B, and C represent the RLG gain area, while areas F, G, and H represent the RLG nongain area.

## 3. Models of RLG Bias Compensation

### 3.1. Support Vector Machine Theory and Its Application in RLG Bias Compensation

The SVM was first proposed by Vapnik *et al.* in 1995. Similar to the multilayered perceptron neural network (MLPNN) and RBFNN, the SVM can also be used to solve model classification and nonlinear regression problems. The key advantage of the SVM is an optimal hyperplane, which separates the sample data without errors and has the largest distance to the nearest sample data.

For multi-input and single-output applications, the SVM is a better solution for machine learning. Suppose the data sampled from the RLG are the linearly separable training set $(x_{i},y_{i}),i=1,\ldots ,n$, where y={+1,−1} is the classification label. The hyperplane is given by

(1) w⋅x+b=0

where w and b are parameters of the hyperplane, which should correctly classify all the sample data. This plane separates the sample data without errors and has the largest distance to the nearest sample data, which is equivalent to minimizing ${∥w∥}^{2}$. The restraint condition is

(2) $$y_{i}[(w\cdot x_{i})+b]-1\geq 0,i=1,2,\ldots ,n$$

The hyperplane that is satisfied by this condition and minimizes the value of ${∥w∥}^{2}$, is the optimal hyperplane. To construct such an optimal hyperplane, only a small amount of training sample data, which determines the margin, is required, and these training data are called support vectors. The optimal hyperplane can also be expressed as a function to find the minimum value:

(3) $$\min\varphi (w)=\frac{1}{2}{∥w∥}^{2}$$

The problem discussed above is the optimal and general classification function, and the only required information is the inner product operations in the characteristic space where the optimal linear classification problem can be solved. For the nonlinear problem, the criteria of the general linear discriminant function can be used to translate the nonlinear problem into a linear problem in another space, where the optimal or general hyperplane can be found.

Mercer’s condition is described as follows: for an arbitrary asymmetric function K(x,y), it is sufficient and necessary that

(4) ∬K(x,y)φ(x)φ(y)dxdy>0

is satisfied for all φ(x), such that

(5) $$\int \varphi^{2}(x)dx<\infty \;$$

Obviously, this condition is easy to achieve, and if the optimal hyperplane dot products are replaced by the inner products K(x,y), the original characteristic space is transformed into a new characteristic space, where the support vector machine is given by:

(6) $$\begin{array}{l} \max Q(a)=\sum_{i=1}^{n}a_{i}-\frac{1}{2}\sum_{i=1}^{n}\sum_{j=1}^{n}a_{i}a_{j}y_{i}y_{j}K(x_{i},x_{j})\\ s.t.\sum_{i=1}^{n}y_{i}a_{i}=0,\;0\leq a_{i}\leq C,\;i=1,2,\ldots ,n \end{array}$$

where C is a penalty parameter, which is used to determine the punish degree. The corresponding decision function is

(7) $$f(x)=sign\left[\sum_{i,j=1}^{n}a_{i}y_{i}K(x_{i},x_{j})+b\right]$$

In this paper, the RBF is selected as the kernel function with the form $K(x_{i},x_{j})=\exp\left(-∥x_{i}-x_{j}∥/2\sigma^{2}\right)$, where σ is the RBF parameter. The other conditions should remain the same.

The principles of the SVM can be summarized as follows: (1) the input space should be transformed to a high-dimensional feature space through nonlinear mapping selected a priori; and (2) the linear decision surface, *i.e*., the optimal linear hyperplane, is constructed between vectors of two classes with maximal margin.

### 3.2. Particle Swarm Optimization Algorithm

The PSO is a novel evolutionary algorithm (EA) that has been developed in recent years [17]. The PSO was first proposed by Kennedy and Eberhart with the intention of simulating simple social behavior such as grouping of fish or bird flocking. The PSO algorithm utilizes a uniformly random solution as the initial condition and finds the optimal solution through multiple iterative calculations. The quality of the solution is evaluated by a fitness function, similar to that of the genetic algorithm (GA), but the PSO algorithm has simpler calculation rules and does not require the tedious process of crossover and mutation operations required in GA.

The PSO algorithm has some advantages, such as easy realization, high accuracy, and fast convergence, because the global optimization solution is achieved by tracking the currently searched optimal value. In the D-dimensional search space, a swarm includes *N* particles, which are described as $X=[x_{1},x_{2},\cdots ,x_{N}]$; each particle is treated as a point and has a memory function. The trajectory of the particle is adjusted according to its best-visited position and the best position of the whole swarm. The best previous position of the particle is described as $x_{i}={\left[x_{i1},x_{i2},\cdots ,x_{iD}\right]}^{T}$, where i=1,2,⋯,N. The velocity of the particle is represented as $v_{i}={\left[v_{i1},v_{i2},\cdots ,v_{iD}\right]}^{T}$. The best position of particle i at the current iteration is expressed as $p_{i}={\left[p_{i1},p_{i2},\cdots ,p_{iD}\right]}^{T}$, and the best position of the global swarm at the current iteration is represented by $p_{g}={\left[p_{g1},p_{g2},\cdots ,p_{gD}\right]}^{T}$. After finding the best particle and global swarm positions, the velocity and position of the particle are updated using the following equations:

(8) $$\begin{array}{cc} v_{id}(t+1)= & v_{id}(t)+c_{1}r_{1}(p_{id}-x_{id}(t))+c_{2}r_{2}(p_{gd}-x_{id}(t))\\ x_{id}(t+1)= & x_{id}(t)+v_{id}(t+1)\\ v_{id}= & \left\{ \begin{array}{c} v_{\max}\;v_{id}>v_{\max}\\ -v_{\max\;}\;v_{id}<-v_{\max}\\ v_{id}\;\left|v_{id}\right|<v_{\max} \end{array}\right.\; \end{array}$$

where i=1,2,⋯,N; d=1,2,⋯,D; $c_{1},\;c_{2}$ denote the acceleration coefficients, which are non-negative constants; and $r_{1},\;r_{2}$ are random numbers uniformly distributed in [0, 1]. The current velocity and position of particle i are given by $v_{id}(t)$ and $x_{id}(t)$, respectively. The value $p_{id}$ describes the local best position, which is the position of the highest fitness value for particle i individually, while $p_{gd}$ describes the global best position, which is the position of the highest fitness value visited by the swarm.

The PSO cannot explore the global and local space simultaneously; thus, a suitable selection of the inertia weight introduced in the update formula can provide a balance between global and local exploration, resulting in better convergence properties [18]. The updated equation is expressed as:

(9) $$v_{id}(t+1)=\omega (t)v_{id}(t)+c_{1}r_{1}(p_{id}-x_{id}(t))+c_{2}r_{2}(p_{gd}-x_{id}(t))\;$$

where

(10) $$\omega (t)=\omega_{\max}-\frac{\omega_{\max}-\omega_{\min}}{T_{\max}}t=0.9-\frac{t}{2T_{\max}}$$

is the inertia weight, t is the number of iterations, and $T_{\max}$ is the maximum number of iterations.

### 3.3. RLG Bias Compensation Utilizing Multiple-Point Temperature Gradient PSO Tuning SVM Algorithms

**Figure 3:**
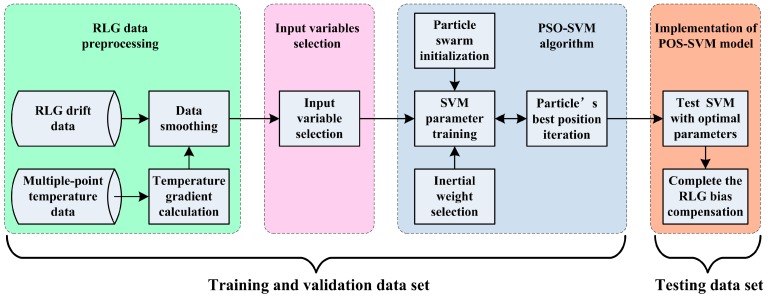
Flowchart of PSO-SVM model in RLG bias compensation.

Because the SVM parameter values have considerable influence on the SVM performance, σ and C in the SVM model should be optimized by the PSO algorithm in order to obtain a higher performing SVM. A flowchart of the PSO-SVM model is shown in Figure 3.

The specific steps of the RLG bias compensation process utilizing the multiple-point temperature gradient with PSO tuning of the SVM algorithm can be constructed as follows:

Step 1. RLG data preprocessing:

(1) Obtain the RLG bias data and multiple-point temperature data for training and validation.
(2) Calculate all of the multiple-point temperature gradients between each of the eight temperature points.
(3) Smooth the RLG bias data and temperature gradient data by averaging each 100-data chunk.
(4) Calculate the correlation coefficients between the RLG bias and all of the temperature gradients.

Step 2. Input variable selection:

When the correlation coefficients between the RLG bias and all of the temperature gradients are implemented, the temperature gradient data with higher correlation coefficients are selected as the input variables in the PSO-SVM model.

Step 3. PSO-SVM algorithm:

(1) Randomly initialize the initial velocity and position of the particle in the limited range.
(2) Optimize σ and C utilizing the PSO algorithm. Each particle must be updated by a set of potential solutions.
(3) Update the weighted iteration formula for the global and local velocity and position by solving Equation (9).
(4) Check the final condition. If the termination condition is met or the maximum iteration number is reached, the search process is complete and the current best individual finding remains as the result. Otherwise, t=t+1, and return to Step 2.

Step 4. Implementation of the PSO-SVM model:

(1) Obtain the RLG bias data and multiple-point temperature data as the testing data set.
(2) Compensate the RLG bias error with the multiple-point temperature gradients by utilizing the optimal POS-SVM model.

## 4. Experiment and Result Analysis

### 4.1. Temperature Experiment Setup

To obtain the data for RLG temperature bias modeling, a temperature cycle experiment is performed. The chamber temperature is arranged with four different rate cycles. In the first cycle, the program is setup as follows: (1) the RLG works under a constant temperature of 20 °C for 2 h to achieve thermodynamic equilibrium; (2) the temperature is decreased at a rate of 1 °C/min until it reaches −40 °C, where it is maintained for 2 h; (3) the temperature is increased at a rate of 1 °C/min to 60 °C, where it is maintained for 2 h; (4) the temperature is decreased by 1 °C/min to −40 °C and is maintained there for 2 h; (5) the temperature is increased by 1 °C/min to 60 °C and is maintained at 60 °C for 2 h; and (6) the temperature is decreased at a rate of 1 °C/min to 20 °C, where it is held for 2 h, completing the first cycle. For the second cycle, the temperature rate of change is set to 2 °C/min, while the other settings are the same as the first cycle. For the third cycle, the temperature rate of change is set to 3 °C/min, while the other settings remain the same. Finally, the temperature rate of change is set to 5 °C/min, while the other settings are the same as those of the first cycle.

**Figure 4:**
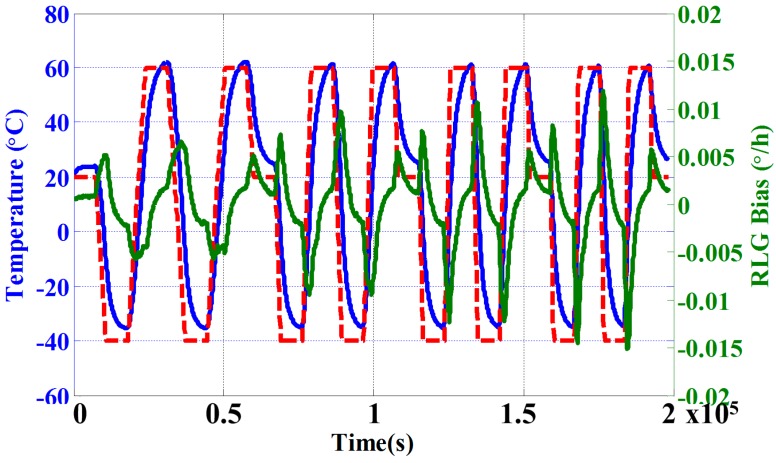
Temperature chamber setting with RLG bias and temperature.

As shown in Figure 4, the dashed red line demonstrates the chamber temperature setting. The solid blue curve is the temperature sampled from the RLG block. The solid green curve shows the RLG bias variation with temperature. In particular, rapid temperature changes result in the worst RLG bias performance.

### 4.2. Correlation Analysis between RLG Bias and Multiple-Point Temperature Gradient

As discussed earlier, the temperature gradient is an important parameter in RLG bias error compensation, but it is still difficult to select the right location for the thermometers. In this study, eight temperature points are considered in the RLG and the cabinet, and correlation analysis is performed between the RLG bias and all of the temperature gradients, which are calculated between each of the eight temperature points. The total correlation coefficients between the RLG bias and the temperature gradient array of eight temperatures are listed in Table 3.

**Table 3:** The correlation coefficients between the RLG bias and the temperature gradient array.

Correlation Coefficients	Thermometers							
	A	B	C	D	E	F	G	H
A	0	0.939	0.938	0.913	0.938	0.912	0.935	0.942
B	0.939	0	0.781	0.888	0.898	0.902	0.913	0.739
C	0.938	0.781	0	0.876	0.633	0.242	0.824	0.916
D	0.913	0.888	0.876	0	0.924	0.831	0.904	0.647
E	0.938	0.898	0.633	0.924	0	0.733	0.412	0.231
F	0.912	0.902	0.242	0.831	0.733	0	0.647	0.750
G	0.935	0.913	0.824	0.904	0.412	0.647	0	0.932
H	0.942	0.739	0.916	0.647	0.231	0.750	0.932	0

**Figure 5:**
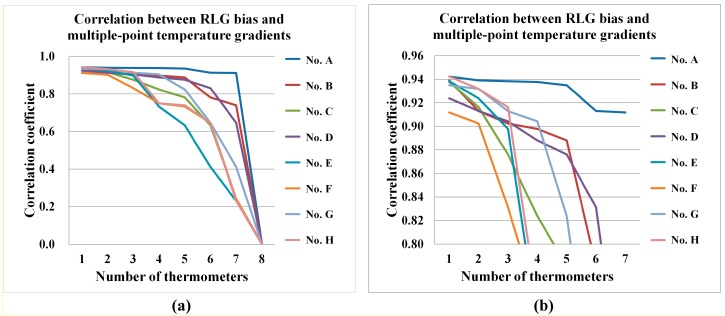
(**a**) Correlation analysis between the RLG bias and multiple-point temperature gradient algorithm; (**b**) Enlarged figure obtained from (**a**).

The correlation analysis between the RLG bias and the multiple-point temperature gradients is shown in Figure 5. As shown in Figure 5, the X-axis shows the number of thermometers, while the Y-axis is the correlation coefficient between the RLG bias and the temperature gradient. The colored lines represent the different thermometers. All seven of the correlation coefficients for the temperature gradients related to thermometer A, which is placed on the surface of the RLG block near the cathode, are greater than 0.90. This result implies that the other temperature gradients, which come from the deviation with thermometer A, have strong relationships to the RLG bias, so compensation with these parameters should be effective. Besides, we can see from Figure 5b (a magnification of Figure 5a), that the two correlation coefficients with areas H and G are also higher than 0.93. This means that the RLG bias is also sensitive to the temperature gradient from the lowest temperature area G and the combined prism area H. This is where the CW (clockwise) and CCW (counter-clockwise) laser beams are combined with a prism and interfere to form a fringe pattern, which is the sample source of the RLG bias [24].

### 4.3. RLG Bias Compensation Results Utilizing Multiple-Point Temperature Gradient PSO Tuning of SVM Algorithm

**Figure 6:**
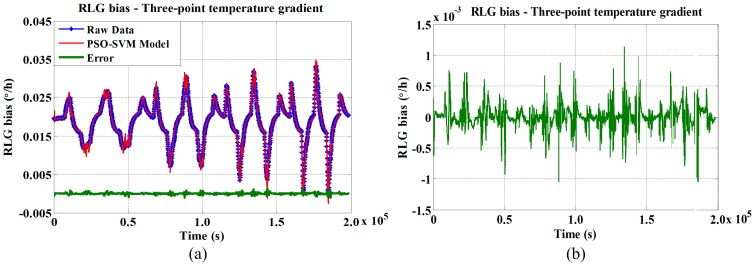
RLG bias compensation model utilizing traditional three-point temperature gradient algorithm at (**a**) different temperature variation rates; and (**b**) enlarged error curve.

**Figure 7:**
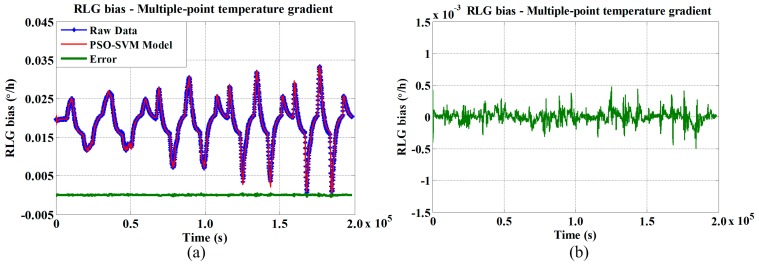
RLG bias compensation model utilizing novel multiple-point temperature gradient algorithm at (**a**) different temperature variation rates; and (**b**) enlarged error curve.

According to Figure 5, we find that the most of the correlation coefficients for temperature gradients related to thermometer A, which is placed near the cathode, are higher than 0.90. Among the other correlation coefficients of temperature gradients with RLG bias, at least two correlation coefficients are greater than 0.90, which means the multiple-point temperature gradients all contribute to the RLG bias variation. To achieve the highest compensation accuracy, the temperature gradients that have correlation coefficients higher than 0.90 are selected as the PSO-SVM model input data set. The compensated results of the RLG bias utilizing the traditional three-point temperature gradient model and the novel multiple-point temperature gradient model are shown in Figure 6 and Figure 7, respectively.

As shown in Figure 6a, the temperature variations in the RLG bias are compensated. However, the enlarged error curve, which is shown in Figure 6b, indicates that the compensation performance decreases when the temperature variation rate increases, especially at inflection points of the temperature.

The compensated results for the RLG bias obtained utilizing the novel multiple-point temperature gradient PSO-SVM algorithm are shown in Figure 7.

In Figure 7, we see that our method effectively compensates for not only temperature variations in the RLG bias, but also accuracy degradation at the inflection point. In order to compare the effectiveness of the proposed method, the standard deviation (STD) estimation method was used as the performance criterion [25]. The standard deviation of the RLG bias $B_{s}$, which is used in this manuscript, is defined as follows:

(11) $$\begin{array}{l} B_{s}={\left(\frac{1}{N-1}\sum_{j-1}^{N}(B_{aj}-\frac{1}{N}\sum_{j=1}^{N}B_{aj}{)}^{2}\right)}^{\frac{1}{2}}\\ B_{aj}=\frac{1}{\tau }\sum_{j=1}^{\tau }B_{i} \end{array}$$

Here, τ is the number in one cluster, $B_{i}$ is the RLG bias sampled at 1 Hz, and N is the number of clusters, which is rounded by the division of the total sample number and the cluster τ. The value of τ in this manuscript was 100 s, which is the same as that in reference [15].

The STD estimations for the original and traditional three-point temperature gradient compensated data are 0.0258 °/h and 0.0089 °/h, respectively; the STD estimations for the original and the novel multiple-point temperature gradient compensated data are 0.0258 °/h and 0.0021 °/h, respectively. Compared with the traditional three-point temperature gradient algorithm, the novel multiple-point temperature gradient algorithm improves the precision of the RLG bias by 40.3%, as shown in Table 4. This result validates the effectiveness of the proposed method.

**Table 4:** RLG bias compensation effect of particle swarm optimization-support vector machine (PSO-SVM) model using different temperature gradient algorithms in temperature variation rate experiment.

RLG Bias (°/h)	Parameters Used in PSO-SVM Model	
	Traditional Three-Point Temperature Gradient	Novel Multiple-Point Temperature Gradient
Before compensation	0.0258	0.0258
After compensation	0.0089	0.0021
Improvement from original accuracy	65.5%	91.9%
Improvement from traditional method		40.3%

**Figure 8:**
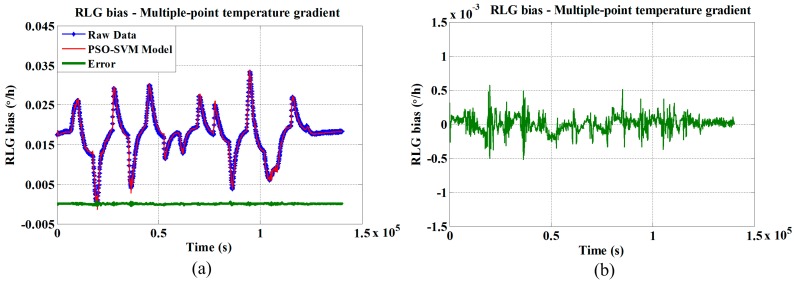
RLG bias compensation model utilizing novel multiple-point temperature gradient algorithm with (**a**) random temperature variation rates, and (**b**) enlarged error curve.

The random temperature experiment is designed to verify the generalizability of the proposed method, and the results are shown in Figure 8. The STD estimations for the original and traditional three-point temperature gradient compensated data are 0.0283 °/h and 0.0091 °/h, respectively; the STD estimations for the original and novel multiple-point temperature gradient compensated data are 0.0283 °/h and 0.0023 °/h, respectively. The novel multiple-point temperature gradient method shows 35.5% improvement over the traditional method (see Table 5).

**Table 5:** RLG bias compensation effect of PSO-SVM model using different temperature gradient algorithms in random temperature experiment.

RLG Bias (°/h)	Parameters Used in PSO-SVM Model	
	Traditional Three-Point Temperature Gradient	Novel Multiple-Point Temperature Gradient
Before compensation	0.0283	0.0283
After compensation	0.0091	0.0023
Improvement from original accuracy	67.8%	91.9%
Improvement from traditional method		35.5%

This experiment shows that the distinct varying trends in the original RLG bias are eliminated, and the algorithm effectively compensates for the temperature drift.

## 5. Conclusions

In this paper, a multiple-point temperature gradient algorithm is proposed for ring laser gyro bias compensation. Based on the multiple-point temperature measurement system, the complete thermo-image of the RLG block is developed. Combined with the temperature and the temperature gradient between the different points of the RLG block, the PSO algorithm is used to tune the SVM parameters, and an optimized design for selecting points for the thermometer is also proposed. The simulation and experimental results validate the superiority of the introduced method, which could be practically applied to enhance the RLG precision. Our future work will be focused on development of data denoising techniques and the fusion algorithm in the navigation system, including three or more RLGs.
